# Grounding the Connection Between Psyche and Soma: Creating a Reliable Observation Tool for Grounding Assessment in an Adult Population

**DOI:** 10.3389/fpsyg.2021.621958

**Published:** 2021-03-08

**Authors:** Einat Shuper Engelhard, Michal Pitluk, Michal Elboim-Gabyzon

**Affiliations:** ^1^Graduate School of Creative Art Therapies, Faculty of Social Welfare & Health Sciences, Emili Sagol Creative Arts Therapies Research Center, University of Haifa, Haifa, Israel; ^2^Graduate School of Creative Art Therapies, Faculty of Humanities & Social Sciences, Kibbutzim College of Education, Tel Aviv, Israel; ^3^Physical Therapy Department, Faculty of Social Welfare & Health Sciences, University of Haifa, Haifa, Israel

**Keywords:** body psychotherapy, dance movement therapy, diagnostic processes, grounding, observation tool

## Abstract

The concept of *grounding* is accepted and common among dance movement therapists and body psychotherapists. It expresses a stable physical and emotional presence – “supported by the ground.” The assumption is that embodied emotional knowledge is expressed through the manner of physical holding and in the emotional experience in the world. However, along with the clinical use of the term, an empirical tool for examining grounding is lacking. The goal of the study was to examine the reliability and validity of an observation tool for assessing the quality of grounding, the Grounding Assessment Tool (GAT), which was created for the present study on the basis of theory, research, and clinical knowledge in the field. Forty three adult participants (age, *M* = 28.2 years, SD = 8.54) were recruited for an experimental and controlled session, the session included guided movement for approximately 10 min. The movement was recorded on video. The quality of the movement was rated by two raters and was scored using the GAT. The study findings indicated that the GAT is a reliable and valid tool – with good internal consistency (α = 0.850) and high interrater and intrarater reliability (Kendall’s ’range from 0.789 to 0.973 and intraclass correlation coefficient range from 0.967 to 1.00, respectively). The exploratory factor analysis showed that four factors are involved in the assessment of grounding quality: fluid and rhythmic movement, emotional expression in movement, pattern of foot placement, and lack of stability and weightiness. The results of this study expand the theoretical understanding of the concept of grounding. They contribute to the understanding of the benefits of body focus, dance and movement in psychotherapy and to validating body psychotherapy and dance movement therapy (DMT). The existence of a reliable and valid tool is essential for assessment and diagnostic processes, for formulating therapeutic goals focused on the body, and for examining their effectiveness.

## Introduction

The concept of *grounding* was first developed by Lowen (1958/1979), one of the first body psychotherapists. Grounding expresses the nature of the psycho-physical presence in the “here and now” in relation to the degree of physical and emotional support by the ground (the ability to be “down to earth,” “feet on the ground”) (Hilton, 2000; Meekums, 2002; Lowen, 2004; Guest et al., 2019). Enhancing grounding by structured movement interventions in conditions of depression (Pylvänäinen et al., 2015), post-trauma (Ko, 2017), and for somatoform disorders (Nickel et al., 2006) are in widespread clinical use among body psychotherapists and dance movement therapists. The assumption is that movement interventions for grounding improvement help to improve the individual’s functioning, emotion regulation and emotional awareness (Guest et al., 2019). Despite the extensive clinical use of the concept of grounding, a comprehensive literature review indicates the lack of an empirical tool for assessment of grounding quality, as well as a lack of research based on experiments and observations in the field. The present study aims to bridge these gaps by creating a reliable and valid tool for examining the contribution of physical aspects of movement to emotional aspects.

The components of grounding embody both physical and emotional aspects. There is agreement among theorists and clinicians that grounding is expressed in the flow quality of movement along the body – meaning, the flow path of movement from the head, through the pelvis, to the feet resting on the ground and from the ground to the head (Guest et al., 2019). According to Lowen (1958/1979), when a person is standing and his body muscles are relaxed (without tension), an energetic flow and discharge of energy into the ground is made possible. In that situations, the movement will be seen as a pulsating wave (i.e., pendular swing) flowing along the body and is evidence of an experience of physical and emotional grounding (Helfaer, 2011).

From Lowen’s description, one can learn about the energetic component of the concept, the same component that connects physiological aspects (such as metabolism and breathing) and emotional processes of grounding (Mahr, 2003; Merriam-Webster, 2014):

“We move by discharge of *energy* [emphasis added] into the ground. All energy finds its way eventually into the earth; this is the principle known as *grounding* [emphasis added]. It explains the discharge through storm and lightening of the overcharged atmosphere” (Lowen, 1958/1979, p. 80.)

The embodiment of the mind as expressed in the concept of grounding is influenced by primary psychomotor developmental processes. The development of equilibrium systems, the tactile system and proprioceptive sense (perception of the position of the body in space) support the baby’s transition from lying to standing and moving in space in a vertical, upright, and stable position with both feet on the ground and both hands free to hold objects (Clauer, 2011). These processes include moving from a horizontal position that expresses, emotionally, dependence on another, to self-holding of the body under the influence of gravity.

In other words, the assumption is that in the face of gravitational forces and interpersonal life experiences, carrying body weight and mobility in space, intensity of muscle use or relaxation and supportive and stable experience alike are all related to previous emotional experiences with the other and accompany the physiological aspects of development. These psycho-physical processes from the beginning of life may be expressed in body possession in adulthood (Hackney, 2002; Clauer, 2011). Body psychotherapists and dance movement therapists invite the patient to deepen awareness of this psycho-physical experience through movement interventions that support balance and stability, and deepen awareness of sensation and organ placement in relation to the other and to space (de Tord and Bräuninger, 2015).

Reich (1933) was the first, even before Lowen, to argue for mind-body unity in Western psychotherapeutic treatment. Reich explored his patients’ body-holding (relationships between body parts, posture changes, and muscle contraction and relaxation actions) while raising emotional issues (Helfaer, 2011). However, while Reich wanted his patients to release muscle holding while lying on a couch during emotional therapy, Lowen favored the use of a vertical standing posture, arguing that for his patients to feel safe, they need to feel the contact of their feet with the ground (Miller, 2010).

The analytical working method that Lowen developed is called *bioenergetic analysis* and involves assessment and intervention in the emotional realm through experiences in the body and movement (Lowen, 2004). The assumption underlying this method is that important life experiences are preserved not only in the mind but also in body posture, movement and breathing, and that physical, mental and emotional processes are intertwined (Nickel et al., 2006). Unlike gymnastic exercises, bioenergetic analysis is directed towards a person’s emotional needs and their expression in the body (Nickel et al., 2006). Accordingly, improving grounding is a fundamental goal in this therapeutic approach (Miller, 2010).

A limited number of experiments have examined grounding’s contribution to emotional processes and included subsequent interventions to improve grounding – breathing exercises, sound output, awareness of body boundaries, and body experiences (Nickel et al., 2006), and experiences of exerting force in a foot to floor encounter (i.e., pushing movement) (Ko, 2017). The findings were that these interventions resulted in a greater reduction in depression among patients compared to a control group of patients who participated in only conventional psychological treatment (Röhricht et al., 2013; Pylvänäinen et al., 2015). A greater decrease in anxiety, depression, and an increase in the ability to regulate the intensity of anger in patients with somatoform disorders compared with patients who participated in physical exercise classes was also found (Nickel et al., 2006). In a qualitative study involving a patient of Korean descent who suffered from abuse, somatic symptoms, and depression, an increase in awareness of body weight, somatic sensations, and mental experiences was reported following physical intervention to strengthen grounding. In all of these studies, the use of grounding was done during emotional therapy and in accordance with the emotional content that emerged from the patients. As mentioned, along with the lack of empirical research, there are many clinical descriptions describing therapeutic work to improve grounding in diverse populations (for a comprehensive review of case descriptions, population and diagnosis, treatment, intervention elements, and consequences of treatment, see Table 1). The interventions include the use of force in the lower body, contraction and relaxation of the muscles, exercises to raise awareness of contact of the feet with the ground, and eye contact with the other while moving and encouraging spontaneous movement (Stromsted, 2007; Miller, 2010; Clauer, 2011; Koch and Harvey, 2012; de Tord and Bräuninger, 2015). In many examples, the therapist’s support for the patient’s psycho-physical grounded presence is based on group work that allows several people to join a common rhythm/movement as a way to raise the sense of unity and self-awareness of each participant (de Tord and Bräuninger, 2015; Karampoula and Panhofer, 2018; Havsteen-Franklin et al., 2019). In these cases, the therapist’s invitation to movement is the outcome of a psychophysical expression of previous experiences that shaped the holding and movement in the patient’s body with the goal of the movement interventions is to bring about a change in the patient’s sense of security, and self-awareness (e.g., a patient who experienced childhood abuse and felt unable to move freely in her lower body).

**TABLE 1:** Clinical Descriptions of Grounding Use.

**References**	**Population and diagnosis**	**Treatment**		**Intervention elements**	**Consequences of treatment**
		**Type**	**Length and frequency**		
Baum, 1997	A middle-aged man with borderline personality organization	Psychotherapy group	Details of treatment length are not described	Pay attention to sensory awareness	Increased self-awareness
Clauer, 2011	A 55-year-old woman with hallucinations, severe depression, dependent-narcissistic personality traits and anxiety	Psychodynamic individual therapy that includes bioenergetic elements	Details of treatment length are not described	Stamp feet lightly on the ground; describe the hallucination more precisely	Reduced anxiety; disappearance of hallucinations
de Tord and Bräuninger, 2015	Between 10 and 15 older people with dementia	DMT open group	Twenty-two sessions over a period of two years, one hour weekly	Grasp an elastic fabric while moving; walk while stamping the feet on the ground; pat body parts; self-massage the body; sound production	Creation of social interaction; increased self-confidence, sense of enjoyment and playful ability; integration between physical experiences
de Tord and Bräuninger, 2015	Three adult women (from 26 to 50 years old) with intermediate intellectual disabilities	DMT group	Twenty sessions over a period of two years, one hour weekly	Walk while stamping the feet on the ground with straps with bells attached to the feet; rub the body using the palms; pat body parts	Posture alignment; improvement in synchronization and physical ability; creation of social interaction
de Tord and Bräuninger, 2015	A 26-year-old woman with intellectual disabilities	DMT individual	Twenty sessions over a period of two years, one hour weekly	Lean on a physiotherapy ball (standing or sitting) when the feet are in contact with the ground	Increase in physical strength, stability, sense of security and ability to be physically and emotionally supported
Koch and Harvey, 2012	An adult woman with dissociative identity disorder, had suffered sexual abuse	Individual DMT	Details of treatment length are not described	Stamp the feet on the ground; develop strength effort; give in to the pull of gravity	Increase in spontaneous movement; reduced anxiety
Stromsted, 2007	A 28-year-old woman with depression, anxiety, panic attacks, and had suffered physical and emotional abuse	Individual DMT	Details of treatment length are not described	Stand on both feet, stamp feet on the ground accompanied by verbal expression	Increase in physical and emotional self-awareness, and in physical and verbal expression ability

*DMT, dance movement therapy.*

### The Present Study

The use of grounding as a means to assess emotional strength and as an intervention method in emotional therapy has been accepted and common for decades. However, an experimental examination of the concept and its uniformity in theoretical and clinical use are lacking. There is no reliable and valid quantitative tool for observing grounding quality which is used for research and clinical diagnosis. The goal of the present study was to test the reliability and validity of an innovative empirical tool for grounding assessment – the Grounding Assessment Tool (GAT). We assume that the existence of this valid and reliable tool will help in clinical work and in establishing future empirical research to examine basic assumptions in the body therapy of the mind.

### The Purposes of the Research

1. To test the reliability of an empirical observation tool for assessing grounding quality (GAT) – internal consistency reliability, interrater reliability and intrarater reliability.
2. To test the content validity of the tool using exploratory factor analysis.
3. To test the discriminant validity of the tool by comparing population groups from different fields of knowledge (physiotherapists, dance movement therapists, and artists).

## Materials and Methods

### Design

This study is a cross-sectional design between subjects, for GAT reliability and discriminant validity testing.

### Participants

Forty-four participants (30 women and 14 men) between the ages of 19 and 67 (*M* = 28.2, SD = 8.54) took part in this study. Participants were recruited using a convenience sampling method through campus advertising and university forums. Among the participants, there were 14 physiotherapy students, 13 dance movement therapy (DMT) students, 11 art students, and 6 who were classified in the “other” category. Inclusion criteria were no orthopedic or neurological impairments, no balance disorders, and no vestibular disorders such as vertigo. Table 2 shows participants’ demographic characteristics with respect to age, gender, and field of study. Of the 44 participants, one participant was removed from the data analysis due to a foot deformity that was not reported at the screening stage.

**TABLE 2:** Participants’ demographic characteristics (*N* = 43).

**Characteristics**	***N***	***%***	***M* (*SD*)**
Age			28.42 (8.52)
Sex			
Female	30	69.8	
Male	13	31.2	
Field of study			
Physiotherapy	14	32.6	
Dance movement therapy	13	30.2	
Art	10	23.3	
Other	6	14	

*SD, standard deviation.*

### Measures

#### Personal Details Questionnaire

Self-report questionnaire regarding socio-demographic status and medical history included information such as: age, sex, medications, background illnesses, and education.

#### Grounding Quality Assessment Scale – Grounding Assessment Tool

The tool was developed by Shuper Engelhard, a dance movement therapist, instructor and researcher, with twenty years of experience in the profession, based on the theory and use of the concept in practice. The tool includes 13 items divided into three categories: (a) gravity attitude (items 1–6: lightness of movement, strength of movement, passive holding of the body, lack of ground support, gradual weight transfer, and foot contact with the floor); (b) movement flow along the body’s vertical axis (items 7–8: movement flow along the body, and muscle tension); (c) interaction with the other and the environment (items 9–13: gap between facial and bodily expressions, ability to share emotion, shared rhythm, use of space, and eye contact). Each of the items is rated on a 4 likert-type scale ranging from 1 (*very low level of grounding*) to 5 (*very high level of grounding*) (see Table 3). The overall score of the tool is the item average, and the score range is between 1 and 5.

**TABLE 3:** Grounding Assessment Tool (GAT).

**Item number**	**Definition**	**Description**	**Score**		
			**1**	**3**	**5**
1	Lightness of movement (light weight)	During movement - using the muscles in a way that is supported by the center of the body and creates light and continuous movement “like a ballerina,” without much effort in energy. Supported by full inhalation and exhalation during breathing. Exists in certain organs, not necessarily in the whole body (powerful movement is possible - strong weight, in another organ alternately/in parallel)	No presence of light weight	A single presence of light weight or instances of light weight only following joining the movement of another	Many times the movement looks light, sculptural (movement with joints in different planes: vertical/horizontal/sagittal) and pleasurable
2	Strength of movement (strong weight)	During movement - using the strength of the body in a way that symbolizes presence, without much effort, rigidity and contraction. Supported in the center of the body and by filling up and emptying the lungs during breathing. Exists in certain organs, not necessarily in the whole body (light movement is possible - light weight, in another organ alternately/in parallel)	No presence of strong weight	A single presence of strong weight or instances of strong weight only following joining the movement of another	Many times the movement looks powerful, like a tribal dance
3	Passive holding of the body	While walking in space - a movement characterized by the body sinking downwards, without the support of the center of the body, the body organs are drooping (like a rag doll)	Over 70% of the time	Between 30% and 70% of the time or in a single limb but prominently and throughout the entire movement	Up to 30% of the time
4	Lack of ground support	Walking characterized by the growth of the body upwards, dominant holding in of the chest and stopping breathing, without relaxation of the weight in the lower part of the body	Over 70% of the time	Between 30% and 70% of the time	Up to 30% of the time
5	Gradual weight transfer	Pattern of foot placement while walking, from the heel to the tips of the toes. Assessed in situations of very fast/slow walking	While walking the entire foot is in contact with the ground as one unit more than twice	While walking the entire foot is in contact with the ground as one unit once/twice	While walking the heel is in contact with the ground and then the entire foot is in contact with the ground
6	Foot contact with the floor	Pattern of the entire foot placement while walking	While walking, parts of the foot are not in contact with the ground more than twice (e.g., tips of the toes in the air). The foot may be placed on the ground and immediately afterwards there will be parts that will cut off contact with the ground for a short time	While walking, parts of the foot are not in contact with the ground once/twice	While walking, the heel is in contact with the ground and then the entire foot is in contact with the ground
7	Movement flow along the body	During free dance, a movement travels along the length of the body, from the feet through the center of the body to the head and back, without stopping	Up to 30% of the time	Between 30% and 70% of the time	Over 70% of the time
8	Muscle tension	During free dance, transition between the contraction of the muscles over time and their relaxation, which leads to freedom and flow in movement, with respect to limbs that are moving	Contraction and stiffness in a particular limb along the movement or throughout the body over long periods of time	Transition between stiffness and long sections of tension-free movement	Lack of expression of tension in movement, many transitions between contraction and relaxation, and flow in movement
9	Gap between facial and bodily expressions	Variation of affect, and coordination between the quality of movement (movement distribution, intensity of movement, connection to the other) and facial expression (contraction and relaxation)	Limited expression of affect	Half the time the affect is limited or poor	Variation of affect that expresses changes in movement
10	Ability to share emotion	In a joint movement in a circle - expression of emotion is shared with the other, in a way that the other sees the emotion of the mover	Lack of emotion sharing in movement	Single emotion sharing once/twice	Ongoing/diverse sharing of emotions with the other
11	Shared rhythm	The ability to suitably join in a common rhythm with external music and with the other	Failure to join an external rhythm	Join after search and trial	The mover easily joins an external rhythm and manages to maintain it over time
12	Use of space	The distance between the mover and the other movers is flexible and varies in relation to the position of the other participants (does not invade the other’s space or surprise the other)	Most of the time there is a stagnation in one place in the room or there is a movement that is not adapted to another	There is movement according to the instruction or a single movement that is adapted to another or there is movement that is not adapted after once/twice	The mover is positioned in several areas of the room and initiates a change of position spontaneously and adapted to the other
13	Eye contact	Makes eye contact with the members of the group moving together	Without eye contact or a single glance	Makes eye contact twice for short moments	Continuous eye contact or more than 3 times

### Procedure

Participants who responded to the invitation to participate, and who met the inclusion criteria in the study, were recruited for a one-hour session. The appointment took place in a hall empty of objects at the institution where the researchers are employed. Three to four participants were invited to each session except for a single group of five participants. The size of the hall was 20 m^2^ (65 square feet). The size of the groups was determined according to the ability to capture high-quality video footage of the movement in all parts of the room.

The session included filling out a personal details questionnaire and moving around the room with a group in regular order. The duration of the movement was 10 min in the following sequence: (1) walking in the space (for 3 min). The instruction was initially to “walk normally in the space, as I always walk”; after a few moments, the participants were instructed to “look for new routes in the space,” then to “increase the pace of walking” and finally, to “slow down”; (2) spontaneous personal free movement in the space (for 3 min). First, an instruction was given to “look for movement that begins in the body” and “you can imagine inner music,” then to “let the movement move along the whole body” and finally, “move with this movement in the space and try to change position in the space”; (3) movement at different rhythms in a circle together with the rest of the group participants (for 4 min). First, an instruction was given, saying “we will walk in place in a circle and try to synchronize with the rhythm.” Then, routine rhythmic music without lyrics was played with the instruction to “synchronize with the music rhythm,” and finally, the instruction given was “each one will suggest a movement and the others will synchronize with that movement.” All participants received the same instructions for movement. The guidelines for movement are based on ways of examining the grounding as described in the literature review and include various options for viewing the quality of the movement (alone and together, standing and moving in space, with open/structured guidance). The entire movement sequence of the group was recorded on a video camera.

Two dance movement therapists, recognized as expert raters, with over 10 years of experience watched the videos once for each participant’s ranking and independently ranked the participants’ grounding quality according to the GAT. The raters have qualified as dance movement therapists at different institutions and do not work together. They both ranked all 44 participants for the interrater reliability. Rater A rated the same videos of 20 participants again, 30 days after giving the first scores. These 20 participants (45.45%) were randomly sampled by age, sex, and field of study to reflect the composition of the full study sample and constituted the sample for intrarater reliability.

### Data Analysis

Participants’ demographic data were processed by descriptive statistics to present participants’ basic characteristics. Three measures of GAT reliability were measured: (a) internal consistency reliability; (b) interrater reliability; (c) intrarater reliability. Internal consistency reliability was analyzed using Cronbach’s alpha (α). The acceptable alpha value should be between 0.70 and 0.90 (de Vet et al., 2011). Alpha value lower than 0.70 indicates a low internal connection between the tool items, and an alpha value higher than 0.90 may imply redundancy of items. The correlation between each item and the total score (item-total correlation) should be above 0.30 to demonstrate that the item correlates to the feature that the tool measures. The contribution of each item to the total score was examined using Cronbach’s α value when each item was removed (Cronbach’s α if item deleted). Interrater reliability was analyzed using Kendall’s tau-b correlation coefficients. The GAT items are ranked according to an ordinal scale and therefore a non-parametric test was performed to assess the level of concordance between the raters’ ranking for each tool item. A correlation coefficient of −1.00 indicates complete lack of correspondence, while a value of 1.00 indicates complete correspondence (Abdi, 2007). In addition, the percentage of participants for which there was a percent concordance on the raters’ rankings for each item in the GAT was calculated. Intrater reliability was analyzed using a 2-way mixed effects model, an average-measure intraclass correlation coefficient (ICC). A correlation greater than 0.70 is recommended as a minimum coefficient for reliability (Terwee et al., 2007), and a value greater than 0.75 represents good reliability (Koo and Li, 2016). In addition, the percentage of participants for which there was a percent concordance on the rater’s rankings for each item in the GAT was calculated.

Content validity was tested by an exploratory factor analysis in order to identify the factors that consisted of related variables with a common basis. The analysis was performed on 13 GAT items using the varimax rotation, according to the criterion of an eigenvalue >1.

The discriminant validity of the tool was examined using a one-way ANOVA to check whether there was a difference in the GAT average score between the groups of participants according to field of study. Tukey’s *post hoc* tests were performed when the ANOVA indicated a significant effect.

The ceiling effect of the tool was measured by calculating the percentage of participants who received the maximum average score in GAT. Presence of a ceiling effect is defined when more than 15% of the participants received the maximum score. This indicates that a population with a high level of the trait measured in the tool is not evaluable, as items at the top of the scale are missing and therefore content validity is limited (Terwee et al., 2007). Data analysis was performed using SPSS 21 software (SPSS, Chicago, IL, United States). Significance was determined by *p* ≤ 0.05.

### Ethics

The study was reviewed and approved by the ethics committee of the University of Haifa, Haifa, Israel, where it was conducted (Approval 2073).

## Results

### Internal Consistency Reliability

Examining the degree of correlation between the 13 items of the GAT demonstrated good internal reliability (α = 0.850). In addition, a moderate correlation was found between each item and the total score (range from 0.262 to 0.692) (see Table 4). Items 3 and 6 were found to be less correlative with the total score (0.262 and 0.350, respectively), but as they did not significantly increase the alpha value (α = 0.852 and α = 0.853, respectively) it was decided not to remove them from the tool.

**TABLE 4:** Correlation between Grounding Assessment Tool (GAT) items and total scores, and Cronbach’s alpha value when item is deleted (*N* = 43).

**GAT items**	**Corrected item-total correlation**	**Cronbach’s α if item deleted**
1	0.574	0.834
2	0.631	0.83
3	0.262	0.852
4	0.54	0.837
5	0.61	0.832
6	0.35	0.853
7	0.692	0.825
8	0.583	0.833
9	0.478	0.841
10	0.605	0.835
11	0.466	0.842
12	0.433	0.843
13	0.406	0.845

*Cronbach’s *α* = 0.850.*

### Interrater Reliability

A Kendall correlation was calculated to test the correlation between Rater A and Rather B ranking for each participant across 13 items and for each item across 44 participants (see Table 5). A strong and significant correlation was found between the raters’ rankings in the range from 0.789 to 0.973 (*p* < 0.01). It was also found that the percentage of participants for whom there was complete correspondence in the raters’ ranking across the 13 GAT items ranged from 75 to 100%.

**TABLE 5:** Kendall correlation and percent concordance between raters’ ranking for Grounding Assessment Tool (GAT) scores (*n* = 20).

**GAT items**	**Kendall’s tau-b correlation coefficients**	**Percent concordance**
1	0.934**	97.674
2	0.918**	93.023
3	0.853**	88.372
4	0.789**	86.046
5	0.962**	95.348
6	0.951**	95.348
7	0.973**	97.674
8	0.887**	86.046
9	0.805**	86.046
10	0.936**	95.348
11	0.856**	90.697
12	0.941**	95.348
13	0.858**	93.023

**p < 0.01.

### Intrarater Reliability

The ICC between rater A’s rankings for GAT items and the percentage of participants for whom there was complete correspondence in the rater’s rankings was calculated to test tool stability over time (see Table 6). As can be seen in the table, an excellent correlation was found between GAT item scores in the range from 0.967 (95% confidence interval [CI] [0.916, 0.987]) to 1.00. It was also found that the percentage of participants for whom there was complete correspondence in the rater’s rankings ranged from 85 to 100%.

**TABLE 6:** Intraclass correlation coefficient and percent concordance in rater A rankings for Grounding Assessment Tool (GAT) Scores (*n* = 20).

**GAT items**	**ICC [95% CI]**	**Percent concordance**
1	0.993 [0.983, 0.997]	95
2	1	100
3	0.993 [0.983, 0.997]	95
4	0.967 [0.916, 0.987]	80
5	0.984 [0.961, 0.994]	95
6	0.995 0[0.988, 0.998]	95
7	0.985 [0.963, 0.994]	90
8	0.983 [0.958, 0.993]	85
9	0.993 [0.983, 0.997]	95
10	0.991 [0.977, 0.996]	95
11	0.991 [0.977, 0.996]	95
12	1	100
13	0.991 [0.977, 0.996]	95

*ICC, intraclass correlation coefficients; CI, confidence interval.*

### Exploratory Factor Analysis

An exploratory factor analysis was performed on 13 GAT items and showed that the GAT consists of four factors which together explained 72.29% of the total variance. The load pattern and explained variance percentage of each factor are shown in Table 7. All items showed a loading of at least 0.49 on their main factor and a loading of at most 0.50 on the secondary factors. Based on the loadings and item content, the four factors can be described as fluid and rhythmic movement (eigenvalue = 4.87), emotional expression in movement (eigenvalue = 1.89), pattern of foot placement (eigenvalue = 1.53), and lack of stability and weightiness (eigenvalue = 1.11).

**TABLE 7:** Loadings of Grounding Assessment Tool (GAT) factors after varimax rotation and the explained variance percentage for each factor.

	**Factor loading**			
**GAT items**	**1**	**2**	**3**	**4**
Factor 1: Fluid and rhythmic movement				
1. Lightness of movement	**0.691**			0.422
2. Strength of movement	**0.671**			
7. Movement flow along the body	**0.901**			
8. Muscle tension	**0.669**			0.369
11. Shared rhythm	**0.670**			
Factor 2: Emotional expression in movement				
9. Gap between facial and bodily expressions		**0.831**	0.327	
10 Ability to share emotion		**0.804**		
13. Eye contact		**0.779**		
Factor 3: Pattern of foot placement				
5. Gradual weight transfer		0.310	**0.826**	
6. Foot contact with the floor			**0.891**	
Factor 4: Lack of stability and weightiness				
3. Passive holding of the body				**0.722**
4. Lack of ground support		0.500		**0.709**
12. Use of space	0.389		0.396	**0.491**
**Explained variance percentage**	37.442	14.532	11.788	8.534

*Each item’s highest loading is presented in boldface. Factor loadings below 0.30 are excluded.*

High Cronbach’s alpha values were found for the factors: fluid and rhythmic movement (α = 0.839), emotional expression in movement (α = 0.810), and pattern of foot placement (α = 0.804). The alpha value for the lack of stability and weightiness factor was moderate (α = 0.587). The correlation between item 12 (use of space) and the lack of stability and weightiness factor was found to be weaker than the other items, but since its removal did not significantly increase the alpha value (α = 0.489) it was decided not to remove it from the tool. Pearson correlations between the factors are shown in Table 8. Significant moderate correlations were found between the four factors, except for the correlation between the factors of emotional expression in movement and lack of stability and weightiness, which was found to be low and not significant.

**TABLE 8:** Descriptive statistics and correlations for Grounding Assessment Tool (GAT) factors.

**Factors**	**M**	***SD***	**GAT total score**	**1**	**2**	**3**	**4**
1. Fluid and rhythmic movement	3.54	1.114	0.862**	1			
2. Emotional expression in movement	4.224	0.964	0.669**	0.446**	1		
3. Pattern of foot placement	3.26	1.677	0.660**	0.317*	0.381*	1	
4. Lack of stability and weightiness	4.147	0.934	0.689**	0.519**	0.224	0.345*	1

*p < 0.05. **p < 0.01.

### Discriminant Validity

A significant difference was noted in the GAT mean score between the four groups [*F* (3, 39) = 3.535, *p* < 0.05]. A Tukey’s test for *post hoc* analysis demonstrated the group of DMT students (*M* = 4.36, SD = 0.73) received a higher GAT mean score compared to group of physiotherapy students (*M* = 3.47, SD = 0.89) (*p* < 0.05). That is, the grounding quality of DMT students is higher than that of physiotherapy students. Similarly, the difference between the score of DMT students group and the score of art studies students group approached significance (*p* = 0.07).

### Ceiling Effect

Nine percent of participants received a maximum GAT mean score, indicating that the GAT did not demonstrate a ceiling effect.

## Discussion

Over the years, extensive literature has addressed the concept of grounding and its clinical use. To date, however, no reliable and valid tool has been created for empirical assessment of grounding quality. To bridge this gap, the present study included the creation and validation of the GAT. The present research findings demonstrate that the tool has good internal consistency, high interrater reliability and intrarater reliability, and that the tool is valid. Four main factors of grounding were found. The level of grounding quality was demonstrated to be higher among DMT students than among students from the other fields of study. In addition, no ceiling effect was found for the tool. The discussion will amplify the contribution of the research results to understanding the body’s and movement’s contribution to emotional therapy and will clarify the importance of the GAT and its unique contribution to research needs and for clinical use.

### Grounding Assessment Using Observation Tools

The creation of an observation tool requires an examination of its reliability in order to provide information about the amount of error inherent in each score, when the total measurement error determines the validity of the research scores (Kottner et al., 2011). In the present study, the correlation between the created tool items (internal consistency reliability) was found to be good (α = 0.850). This verifies that all items in the tool measure the same theoretical variable and there is no redundancy of items (de Vet et al., 2011).

In addition, a strong correlation was found between the various rater assessments of the participants (Kendall’s tau-b range from 0.789 to 0.973). This indicates that the difference between the participants’ scores is mainly due to the difference in their performance in the experiment, i.e., the differences are due to grounding quality and not to differences in the rater assessments. In addition, the stability of the GAT over time (ICC range from 0.967 to 1.00) indicates high tool intrarater reliability under repeated similar assessment conditions. Inconsistencies and variability in a rater’s assessment may result from intervening variables associated with the rater’s condition, such as fatigue, mood, and level of attention over time, but not from differences in a subjects’ performance (Moskal and Leydens, 2000). In the present study high interrater and intrarater reliability strengthens the effectiveness of the tool.

Grounding quality assessment during the intervention process by psychologists (Turkus and Kahler, 2006; Gendlin, 2012; van der Kolk, 2014; Sar et al., 2017), mind-body therapists (Reich, 1933; Lowen, 2004; Röhricht et al., 2013; Geuter, 2016), and dance movement therapists (Stromsted, 2007; Koch and Harvey, 2012; Ko, 2017) has been performed over the years without the use of valid and reliable observation tools. In light of this study’s findings, the GAT is proposed as a valid and reliable tool that will strengthen the reliability of a grounding assessment process, via a brief 10-min observation, without the need for a verbal interview or the use of physical measurement tools. In addition, the GAT has been demonstrated in this study to be a reliable tool that encompasses the various parameters included in the theoretical concept (body position in relation to gravity, movement flow manner along the body’s vertical axis, and the relationship in movement with the other and the environment) (Guest et al., 2019).

### The Four Factors of Grounding

In order to further investigate the grounding components, an exploratory factor analysis of the tool was performed and indicated the GAT measure’s four main factors: *fluid and rhythmic movement*, *emotional expression in movement*, *pattern of foot placement*, and *lack of stability and weightiness*, expanding the theoretical understanding of the concept of grounding. The *fluid and rhythmic movement* factor refers to the degree of lightness and force mobilization used by the muscle during movement, the flow of movement along the body from the head to the feet, the ability to transition between increasing organ contraction and relaxation (without fixation on contraction or relaxation), and adjustment to a shared rhythm. This factor, as part of the GAT assessment, is consistent with the basic assumption of the grounding theory proposed and developed by Lowen (1958/1979), who argued that the quality of flow movement throughout the body, without areas of tension and contraction, indicates physical grounding. The rhythm factor examines the ability to join an external rhythm, which has been shown to contribute to the ability to be in present awareness of oneself and the environment at a given moment (Bräuninger, 2014a). One of the basic assumptions in DMT is that movement in common rhythm with the patient and joining their movement form produces a sense of group cohesion, security, personal empowerment, experience of belonging, communication and healing (Capello, 2009). Chace (Chaiklin, 1975), one of the founders of the field, frequently emphasized that even symbolic rhythmic actions bring emotional content to consciousness. The present study emphasizes that these qualities are an integral part of the grounding assessment.

The *emotional expression in movement* factor examines the diversity of emotional expression in movement in general and in relation to others, the coordination between the movement quality and facial expressions, and manner of eye contact during joint movement in a group. In the 19th century Darwin (1872/1965) observed that emotions are reflected in movement and facial expressions. Since then, extensive clinical literature has described the relationship between emotional expression and the patient’s sensory, physical, and emotional awareness in emotional therapy (de Tord et al.,2015; Stromsted, 2007; Clauer, 2011). As stated, the present study emphasizes the importance of this factor in grounding observation which aims to assess an emotional and physical experience, a regulated, conscious and stable presence, both physical and emotional.

The *pattern of foot placement* factor, which examines the manner in which the foot is spread on the floor, refers both to all parts of the foot being in contact with the floor and the gradualness with which the different parts of the foot come in contact with the floor while transferring weight from heel to toe when walking. This is in line with Lowen’s theory that a grounded person is a person with their feet on the ground and that the grounding level corresponds to the degree to which the foot fully touches the ground (Lowen and Lowen, 1977). In a previous study, it was found that the pattern in which the foot is positioned when walking is critical for maintaining physical stability (Bruijn and van Dieën, 2018). A normal walking model includes striking the heel on the ground followed by the toes, as the walking phase progresses lifting the heel off the ground precedes lifting the toes (Anwary et al., 2018). Also, assuming that stability is related to the gradual spreading of the foot on the ground, it was found that typical walking is characterized by the transfer of the center of body weight from the heel to the toes contributes to control of vertical posture (Gruben and Boehm, 2014). That is, studies that use physiological metrics reinforce the assumptions embodied in the concept of grounding and clarify the importance of scoring this factor.

It is interesting to note that in the present study, differences between participants in the pattern of foot placement were manifested only at the time of instruction to accelerate the participants’ natural walking pace or to slow down movement. An invitation to get out of the safe and familiar rhythm, confronts the movers with abilities at the pre-effort level. Pre-effort is a term coined by Kastenberg (Amigi et al., 1999), which describes movement qualities that appear when confronted with a new and unknown task. According to Kastenberg, the movement quality in pre-effort situations is based on internal orientation (fears/anxieties, insecurity in the face of a task), and is associated with high awareness of the self and the environment, as opposed to actions performed automatically (Koch, 2007; Roberts, 2016). The results of the present study validate this aspect in Kastenberg’s assessment profile.

The factor *lack of stability and weightiness* includes a reference to the degree of passivity in body holding, lack of ground support, and the manner of movement in space while taking into account the other’s location. This is in line with the assumption in the literature that grounded motion under the influence of gravity requires carrying the body’s weight and changing it while moving in space in order to maintain physical stability (Hackney, 2002; Clauer, 2011). The understanding that the assessment of lack of stability and weightiness is separate from the assessment of movement flow emphasizes the importance of addressing movement observation both to the strengths expressed in body use and to the lack of forces in the organization of the moving person in the face of gravity and the environment.

### Discriminant Validity of the GAT

The current study involved 44 students from three disciplines: physiotherapy, DMT, and art studies. Examining the differences between the groups in the GAT score, a trend was found that indicates a higher level of grounding quality among DMT students as compared to the other groups. This may be related to the fact that DMT uses grounding interventions, reflective thinking, and training that increases physical and emotional awareness. Federman (2011) found that the kinesthetic abilities (conscious perception of movement, sensation and balance) of DMT students increased during their academic training compared to art therapy students.

It should be noted that the GAT has no ceiling effect. Therefore, the level of grounding quality can be measured using the tool even in a population with a high grounding level (Terwee et al., 2007), as demonstrated in the current study with the group of DMT students, and it can distinguish between participants within the same population and between it and other populations.

## Conclusion and Practical Implications

The present study proposes the GAT as a reliable and valid tool for assessing grounding quality among healthy subjects in a brief 10-min observation. The results of the study indicate that grounding assessment according to the tool, by therapists with at least 10 years of experience, yields a high correlation between the tool items, between different raters and for the same rater (intrarater). The study shows that grounding quality assessment includes four factors: fluid and rhythmic movement, emotion expressing in movement, pattern of foot placement, and lack of stability and weightiness. To score the quality for each factor, observing the movement while walking and dancing freely is required, when the person is attentive to their natural rhythm and when they are invited to accelerate or decelerate the rhythm according to external instructions, or when following external music, while interacting with other participants. The results of this study expand the theoretical understanding of the concept of grounding and contribute to understanding the benefits of body focus, dance and movement in psychotherapy, and to validating the field of body psychotherapy and DMT. Through further research that will make use of GAT before and after the intervention, the GAT may help diagnostic and assessment processes and to understand the effectiveness of basic clinical interventions in DMT and body psychotherapy. The GAT, which examines grounding quality, can serve as a springboard for future research in the fields of body therapy and DMT with diverse populations and emotional difficulties.

## Limitations and Further Studies

From the findings of the present study, it can be seen that the level of grounding quality was greatly affected by the “fluid and rhythmic movement” factor. In addition, the strong relationship between the factors of “fluid and rhythmic movement” and the “lack of stability and weightiness” indicate that the greater the flow in movement, there are fewer manifestations of lack of weight. These findings are correct for a mostly young adult population. It is possible that in other populations another factor has a stronger influence, and different strong relationships will be found between the factors. Therefore, the number of factors in the tool has not been reduced and we offer further studies that will examine the factors’ ability to diagnose and their relationships.

Due to the nascency of the field and the complexity of the observations and their scoring, the present study was performed on a small sample. In further studies, a larger sample size will make it possible to examine whether the current results that conformed to the direction of the hypotheses but were not statistically significant will be found to have statistical significance. Additionally, the study population included a majority of young healthy subjects, most of whom were students living in a Western culture. Accordingly, it is recommended in future studies to assess grounding quality in diverse populations in terms of age, culture, and the existence of physiological and psychological pathologies. Various studies have found that the emotional expression ability of children and non-Western cultures is different from that of the older and Western population (Steinberg et al., 2006; Kim and Sherman, 2007). Examining the GAT in these populations can contribute to a further understanding of grounding and its emotional aspect.

In addition, the study was conducted in an enclosed space suitable for the movement of up to five participants, and the room size may have affected the participants’ performance in the experiment. The current room size may explain the finding of a weaker connection between the space use item and the lack of stability and weightiness factor than between the other items associated with this factor. Further research should examine the effects of space conditions on movement (such as larger spaces or open spaces) and on grounding quality.

In further research, the tool validation should be further examined by comparing the ranking of dance movement therapists in different countries. The tool reliability should also be examined in relation to self-report questionnaires and to physical measurement tools for examining body stability.

## Data Availability Statement

The raw data supporting the conclusions of this article will be made available by the authors, without undue reservation.

## Ethics Statement

The studies involving human participants were reviewed and approved by University of Haifa, Research Authority. The patients/participants provided their written informed consent to participate in this study.

## Author Contributions

ES initiated and designed the study. MP helped to write the manuscript and analyzed the data. MG-E brought her research expertise in the assessment and treatment of balance and gait capabilities and assisted in design the study. All authors contributed to the article and approved the submitted version.

## Conflict of Interest

The authors declare that the research was conducted in the absence of any commercial or financial relationships that could be construed as a potential conflict of interest.
